# Standardized Delirium and Dementia‐Friendly Mobile Comfort Carts to Increase Senior‐Friendly Non‐Pharmacologic Care

**DOI:** 10.1002/alz70858_104091

**Published:** 2025-12-25

**Authors:** Justin Y Lee, Matteen Pezeskhi, Arianna R Paolone, Christina Sanders, Susy Marrone, Shari Duxbury, Patricia Ford, Leanne Nightingale, Joye St. Onge, Heather McLeod, Micheline Gagnon, Michele Patterson, Shawn Mondoux, Jane Loncke, Donna Johnson

**Affiliations:** ^1^ McMaster University, Hamilton, ON, Canada; ^2^ Centre for Integrated Care, St. Joseph's Health System, Hamilton, ON, Canada; ^3^ Geras Centre for Aging Research, Hamilton, ON, Canada; ^4^ St. Joseph's Health System Center for Integrated Care, Hamilton, ON, Canada; ^5^ Royal College of Surgeons in Ireland, Dublin, Dublin, Ireland; ^6^ St. Joseph's Healthcare Hamilton, Hamilton, ON, Canada

## Abstract

**Background:**

Hospital environments can be harmful to older adults and contribute to hospitalization‐associated disability. To improve health outcomes, guidelines recommend more emphasis on non‐pharmacological care to better address physical, emotional, cognitive, and rehabilitative needs of older adults. We sought to develop, implement and evaluate the use of standardized mobile ‘Comfort Carts’ containing non‐pharmacological resources to enhance patient comfort, increase cognitive and physical stimulation, and help manage responsive behaviours associated with delirium and dementia.

**Methods:**

A ‘Comfort Cart’ containing a standardized set of non‐pharmacological resources for hospitalized older adults at risk of delirium and responsive behaviours associated with dementia was developed through multidisciplinary stakeholder consultations and a needs assessment survey of clinicians, patients, and hospital volunteers. Hospital volunteers were trained to use the ‘Comfort Cart’ to facilitate patient interactions and non‐pharmacological care to all patients admitted to an orthopedic unit at a tertiary care academic hospital. We conducted daily program audits over a 1‐year period and post‐implementation surveys to evaluate its effectiveness and acceptability.

**Results:**

The ‘Comfort Cart’ program has facilitated over 669 patient visits to date by hospital volunteers, including 50 with patients with dementia or those at high‐risk for delirium and/or responsive behaviours. Over 168 hours of patient‐volunteer interactions were logged with a mean visit duration per patient of 16.5 (SD 19.5) minutes. More than half of visits (58.1%) improved patients’ mood or behaviour. The longer the duration of a visit, the higher the likelihood that it would improve a patient's mood or behaviour. In post‐program implementation surveys, 76% of healthcare staff agreed that the ‘Comfort Cart’ addressed barriers for implementing non‐pharmacological care and 80% of volunteers stated that the ‘Comfort Cart’ helped them have meaningful visits with patients. All patients and caregivers surveyed felt that the program was important to meet the needs of hospitalized older adults, and 91% reported that it positively contributed to the perceived level of compassionate and overall patient care that they received in hospital.

**Conclusions:**

Standardized mobile ‘Comfort Carts’ satisfy unmet non‐pharmacological needs in the care of older adults and positively impact patient experience in hospital.

